# Evaluation of the microbiome in children’s appendicitis

**DOI:** 10.1007/s00384-016-2639-x

**Published:** 2016-09-09

**Authors:** Martin Salö, Nittaya Marungruang, Bodil Roth, Tiia Sundberg, Pernilla Stenström, Einar Arnbjörnsson, Frida Fåk, Bodil Ohlsson

**Affiliations:** 1 Department of Clinical Sciences, Pediatrics, Lund University, Lund, Sweden; 2 Department of Pediatric Surgery, Skåne University Hospital, Lund University, Lasarettsgatan 48, 221 85 Lund, Sweden; 3 Food for Health Science Centre, Lund University, Medicon Village, 22381 Lund, Sweden; 4 Department of Clinical Sciences, Division of Internal Medicine, Skåne University Hospital, Lund University, 205 02 Malmö, Sweden

**Keywords:** Appendicitis, Microbiome, Children

## Abstract

**Background/aim:**

The role of the microbiome has been widely discussed in the etiology of appendicitis. The primary aim was to evaluate the microbiome in the normal appendix and in appendicitis specifically divided into the three clinically and histopathologically defined grades of inflammation. Secondary aims were to examine whether there were any microbiome differences between proximal and distal appendices, and relate the microbiome with histopathological findings.

**Methods:**

A prospective pilot study was conducted of children undergoing appendectomy for appendicitis. The diagnosis was based on histopathological analysis. Children with incidental appendectomy were used as controls. The proximal and distal mucosa from the appendices were analyzed with 16S rRNA gene sequencing.

**Results:**

A total of 22 children, 3 controls and 19 appendicitis patients; 11 phlegmonous, 4 gangrenous, and 4 perforated appendices, were prospectively included. The amount of *Fusobacterium* increased and *Bacteroides* decreased in phlegmonous and perforated appendicitis compared to controls, but statistical significance was not reached, and this pattern was not seen in gangrenous appendicitis. No relation could be seen between different bacteria and the grade of inflammation, and there was a wide variation of abundances at phylum, genus, and species level within every specific group of patients. Further, no significant differences could be detected when comparing the microbiome in proximal and distal mucosa, which may be because the study was underpowered. A trend with more abundance of Fusobacteria in the distal mucosa was seen in appendicitis patients with obstruction (25 and 13 %, respectively, *p* = 0.06).

**Conclusion:**

The pattern of microbiome differed not only between groups, but also within groups. However, no statistically significant differences could be found in the microbiome between groups or clinical conditions. No correlation between a specific bacteria and grade of inflammation was found. In the vast majority of cases of appendicitis, changes in microbiome do not seem to be the primary event. Since there seem to be differences in microbiome patterns depending on the sample site, the exact localization of biopsy sampling must be described in future studies.

## Introduction

Appendicitis is a common disease among children and adults, with a lifetime risk of 7 % [1]. Despite that the first appendectomy was performed over 130 years ago [2], the physiologic function of the appendix and the pathogenesis of appendicitis are not fully understood.

There are several proposed causes behind the development of appendicitis, but the most common explanation to the primary event is an obstruction of the lumen with subsequent accumulation of secretions, rising intraluminal pressure, impairment of the lymphatic and venous drainage, compromised mucosal barrier, and overgrowth and invasion of microbes within the appendiceal wall [3–6]. However, obstruction due to fecaliths, anatomic location, lymphoid hyperplasia, foreign bodies, and tumors is reported to be found only in around a third of all cases [7–9]. The intraluminal pressure was not increased when studied prospectively [7]. In summary, it is clear that the theory with obstruction of the lumen cannot explain the majority of all cases of appendicitis [10], and therefore, the theory of overgrowth and invasion of microbes, secondary to obstruction, is weak. Instead, there are reports indicative of a primary infectious event [11], and one study reported on appendicitis appearing in clusters [12]. There are also reports of a seasonal variation of the incidence of acute appendicitis [13, 14].

Despite the uncertainty regarding the sequence of events leading to the development of appendicitis, it is presumed that the microbiome in the appendix has a central role in the pathogenesis [11, 15–18]. Most previous studies have used conventional culture techniques to evaluate the role of bacteria in acute appendicitis [19, 20]. This technique is effective in evaluating solitary bacterial species, but lacks the capability of characterizing the polymicrobial diversity [11]. With these conventional culturing methods, as much as 90–99 % of the microbes are missed [21]. To evaluate microbial diversity, a 16S rRNA gene-based examination of the appendix microbiota should be carried out [22].

To date, there are five studies using non-culture-dependent methods to characterize the microbiome of the healthy and diseased appendix [11, 15–18], of which two used rRNA-based fluorescence in situ hybridization (FISH) [11, 17] and the other three used a 16S RNA sequencing [15, 16, 18] (Table 1). Swidsinski et al. performed FISH analysis of appendices from different countries, namely Germany [11], and then Russia and China [17]. *Fusobacterium* were not found in any controls, but invasion of *Fusobacterium* was found in the submucosa of the inflamed appendix, and the invasion seemed to increase with the severity of the inflammation [11, 17]. The first study with 16S rRNA sequencing of bacterial DNA from appendices was published in 2013 [15]. In this small study with only seven samples, *Fusobacterium* was found in healthy appendices. However, the highest amount of *Fusobacterium* was found in the inflamed appendices [15]. In addition, also other bacteria found in the oral cavity were increased in the inflamed samples [15]. In the same year, a larger study was published with 16S RNA sequencing from pediatric appendectomy specimens [18]. In analogy, the inflamed appendices were found to have increased abundance of taxa normally found in the oral cavity, i.e., *Fusobacterium*, *Porphyromonas*, *Parvimonas*, and *Gemella*, and reduced the amount of *Bacteroides*, compared with controls [18]. In 2014, Jackson et al. [16] studied the microbiome in appendectomy specimens and rectal swabs from children with and without appendicitis. In normal appendices, *Fusibacter*, *Selonomonas*, and *Peptostreptococcus* were increased compared with normal rectal samples, suggesting a unique microbiome in the appendix. In the inflamed appendices, 12 taxa were significantly increased compared with controls (*Peptostreptococcus*, *Bilophila*, *Bulleidia*, *Fusobacterium*, *Parvimonas*, *Mogibacterium*, *Aminobacterium*, *Proteus*, *Actinomycineae*, *Anaerovorax*, *Anaerofilum*, and *Porphyromonas*), and five taxa (*Bulleidia*, *Fusibacter*, *Prevotella*, *Porphyromonas*, and *Dialister*) were increased in perforated appendicitis compared with non-perforated appendicitis [16]. Interestingly, three taxa increased in the rectal swabs of patients with appendicitis compared with controls (*Bulleidia*, *Dialister*, and *Porphyromonas*) [16].

**Table 1:** Overview of studies of non-culture dependent evaluation of appendicitis

Study	Patients	Method	Results
Swidsinski et al.	52 patients 18 controls	rRNA-based FISH	Invasion of Fusobacterium in the submucosa of the appendix. Fusobacterium not found in any controls and increased with the severity of the inflammation
Swidsinski et al.	86 patients	rRNA-based FISH	
Guinane et al.	4 patients 3 controls	16S RNA sequencing	Highest amount of Fusobacterium found in appendicitis, but Fusobacterium was also found in controls. Gemella, Parvimonas also abundant increased in the inflamed samples.
Zhong et al.	17 patients 5 controls	16S RNA sequencing	Increased abundance of Fusobacterium, Porphyromonas, Parvimonas, and Gemella, and reduced amount of Bacteroides in appendicitis compared to controls.
Jackson et al.	15 patients 6 controls	16S RNA sequencing	Fusobacter, Selonomonas, and Peptostreptococcus increased in normal appendices compared to normal rectal samples. Peptostreptococcus, Bilophila, Bulleidia, Fusobacterium, Parvimonas, Mogibacterium, Aminobacterium, Proteus, Actinomycineae, Anaerovorax, Anaerofilum, and Porphyromonas increased in appendicitis compared to controls. Bulleidia, Fusibacter, Prevotella, Porphyromonas, and Dialister increased in perforated appendicitis compared to non-perforated appendicitis. Bulleidia, Dialister, and Porphyromonas increased in rectal swabs of patients with appendicitis compared to controls.

*FISH fluorescence in situ hybridization, *rRNA* ribosomal ribonucleic acid

In all previous studies, the grade of inflammation was only divided into normal appendix, inflamed appendix, and perforated appendix [11, 15, 16, 18]. Further, sampling of the appendix was different in all studies; luminal fluid [18], swabbing of the appendix [16], whole pieces of appendix [11], and sometimes not fully described [15]. Further, the exact sample site is not described in previous studies. For example, if an obstruction is present in the middle of the appendix, there may be a clear difference in grade of inflammation between the proximal and distal part, and hence, this may affect the microbiome pattern. The primary aim of this prospective study in children was to evaluate the microbiome in normal appendix and in appendicitis specifically divided into the three clinically and histopathologically defined grades of inflammation (i.e., phlegmonous, gangrenous, and perforated appendicitis). Secondary aims were to examine whether there was any microbiome differences between proximal and distal appendices, and relate the microbiome with histopathological findings. In conclusion, what is new in our study is that (a) the mucosa of the appendix was examined, (b) different groups of clinical appendicitis were compared, (c) the microbiome was compared according to histopathological changes, and (d) the microbiome was compared according to localization of the sampling, proximal versus distal appendix.

## Material and methods

The study was performed according to the Helsinki Declaration and approved by the Regional Ethical Review Board (registration number 2013/614) and by the Regional Biobank Center (collection ID SC1956). The data were anonymized prior to calculations and are presented in such a way that it is impossible to identify any single patient. The caregivers were given written and oral information about the study before giving their consent.

### Settings and children

All children were operated at a tertiary center of Pediatric Surgery from August 2013 to July 2014. The center serves an area with 340,000 inhabitants with primary surgical care for children <15 years of age. The diagnosis of appendicitis was based on the clinical examination together with blood tests, and sometimes with the aid of ultrasound. The diagnosis was confirmed by the intraoperative findings and by histopathological analysis. During the study period, six attending surgeons were responsible for the appendectomies. The appendectomy was performed either laparoscopically with two or three ports, or as an open appendectomy with a laparotomy in the right lower quadrant. Patients were included in the study when one of the authors (MS or EA) was on call. For controls, patients with appendectomies during operations for other conditions were used.

### Study design

All data were collected prospectively. The following clinical parameters were registered at the Department of Emergency: age, gender, weight, medication including treatment with antibiotics during the last year, symptoms, value of C-reactive protein (CRP), and Pediatric Appendicitis Score (PAS) [23]. Leukocytes, neutrophils, and high-sensitive CRP were analyzed from venous blood samples at the Department of Clinical Chemistry according to standardized routines.

All children were given the same antibiotic preoperative prophylaxis before the appendectomy with trimethoprim/sulfamethoxazole (16 + 80 mg/ml, dosage according to age; Eusaprim®, Vitaflo Scandinavia AB, Gothenburg, Sweden) and metronidazole (5 mg/ml, 20 mg/kg; Flagyl®, Sanofi AB, Stockholm, Sweden). During the operation, the perioperative grade of inflammation was classified into phlegmonous, gangrenous, or perforated. Gangrenous appendicitis was defined as an inflamed appendix with significant gray or black discolorations of the wall and absence of the criteria for perforation. The definition of perforated appendicitis was a visual hole in the appendix, finding of a fecalith in the abdomen during the appendectomy, or spread purulence within the abdominal cavity [24].

### Tissue sampling

The preparation of the appendix was performed immediately after the appendectomy and carried out by the same surgeon in all children (MS). The length and thickness of the appendix was measured, the distal and proximal 1 cm of the appendix removed, and the appendix cut open with sterile scissors. The mucosa was inspected and presence and distribution of macroscopic inflammation along the appendix, as well as possible obstruction, was noted. The distal mucosa and proximal 2 cm mucosa was scraped off using a sterile scalpel, put into sterile Eppendorf tubes, and immediately frozen. The samples were stored in −80 °C until analyzed.

The rest of the appendix was put in formalin and histopathological examinations were performed by experienced specialists at the Department of Pathology. The outer wall and lumen of the appendix was inspected with regard to obstruction, foreign bodies, purulence, and wall defects. On routine, three sections of the appendix with 3–5 mm thickness was cut out; the base, the middle part, and the tip. If other parts of the appendix had a different gross appearance, sections from this part was also cut out. The histopathological definition of appendicitis was the presence of infiltration of polymorphonuclear neutrophils in the muscularis propria layer [25]. Gangrenous appendicitis was defined as full-thickness necrosis in any of the examined sections [26].

### Analysis of the microbiome

A total of 49 biopsy samples (9 healthy, 23 flegmonous, 7 gangrenous, and 10 perforated) were used in the microbiota analysis. The tissue was thawed on ice and DNA was extracted using the QIAamp DNA Stool Mini Kit (Qiagen), with an addition of a bead-beating step. Sterile glass beads (1 mm) were added in combination with stool lysis buffer and cell disruption was performed for 2 × 2 min at 25 Hz using a TissueLyser (Qiagen), followed by a heating step at 95 ˚C for 5 min. After lysis, DNA-damaging substances and PCR inhibitors were removed using InhibitEX tablet (provided with the kit) and the DNA was purified on QIAamp Mini spin columns.

The V1–V3 regions of 16S rRNA genes were amplified using a limited cycle PCR with forward and reverse primers containing Illumina adapter sequences and dual-index barcodes used for tagging each sample, primer sequences are listed in Table 3. Paired-end sequencing with a read length of 2 × 300 bp was performed on a Miseq Instrument (Illumina, Inc., San Diego, CA) using a Miseq v3 reagent kit (Illumina, Inc., San Diego, California). Sequences were analyzed using Quantitative Insights into Microbial Ecology (QIIME), as previously described [27]. After quality filtering, a total of 36 samples remained and 2,648,892 reads were included for downstream analyses and an average of 71,592 sequences (SD 95,901) were assigned to each sample (ranging from 389 sequences to 461,864 sequences). To correct for sampling depth differences, 4805 reads/sample were randomly selected and utilized for further calculation of alpha-diversity and weighted and unweighted Unifrac, which excluded nine samples from diversity analyses.

### Statistical analyses

Patient data were recorded in an Excel database. Statistical analyses were performed using SPSS (Statistical Package for the Social Sciences). To adjust for possible confounders, we compared the microbiome in the appendicitis patients with regard to gender, using Mann–Whitney *U* test. Further, a Spearman’s rank correlation test was performed between the microbiome in appendicitis patients and age and weight, respectively. When comparing the presence of different phylum and genus at different degrees of appendiceal inflammation with the controls, the Kruskal–Wallis test was used. Comparisons were made including all patients, using the distal analysis when both proximal and distal analyses were at hand. Analyses were also performed between proximal samples and between distal samples. The Wilcoxon-signed test was used to compare phylum and genus levels in proximal and distal samples within each patient. When evaluating differences in the phylum microbiome between appendices with and without an appendicolith, and with or without proximal macroscopic inflammation, Mann–Whitney *U* test was used. Statistical significance was set to a *p* value <0.05.

Regarding the microbiota samples, differences in within-community richness (α-diversity) were calculated in QIIME using a non-parametric *t* test and the *p* value was corrected for multiple comparisons using false discovery rate (FDR) correction [28]. Differences in community composition among groups of samples (β-diversity) were analyzed using the non-parametric analysis of similarity (ANOSIM) [29] statistical test in QIIME on both unweighted and weighted Unifrac phylogenetic metrics. Moreover, linear discriminant analysis (LDA) effect size (LEfSe) analysis [30] was performed to identify differentially abundant bacterial taxa from phylum to species level.

## Results

### Patient characteristics

During the study period, a total of 45 patients with confirmed appendicitis underwent appendectomy. Of these, 27 patients (60 %; 17 males/10 females) were included in the study, with an even distribution over the 12 months. As controls, five patients with healthy appendices collected during operations for other conditions (two with intussusception, two with malrotation, and one intra-abdominal tumor) were also included, resulting in a total of 32 patients enrolled in the study. Every child was of Swedish ethnicity and lived in the same area. All children were healthy prior to the operation and no one used medications on a regular basis. None of the children had used antibiotics within 1 year before the appendectomy. After extraction of DNA and analysis of the microbiome, only the material from 22 patients were sufficient and adequate to analyze; 3 controls and 19 appendicitis patients. Of these, 21 distal mucosa samples and 15 proximal mucosa samples were obtained. In the appendicitis group, 11 patients (58 %) had phlegmonous appendicitis, 4 patients (21 %) had gangrenous appendicitis, and 4 patients (21 %) had perforated appendicitis. An obstruction of the appendix was found in five (26 %) of the appendicitis patients (Table 2). Of the three control patients, two had malrotation and one had an intra-abdominal tumor. Blood parameters reflecting inflammation correlated with the clinically and histopathologically defined grades of inflammation (Tables 2 and 3). No differences in the microbiome between the genders were found, and no correlation between the microbiome and age or weight were seen at phylum or genus level (data not shown). Hence, all patients were calculated together.

**Table 2:** Demographics and clinical parameters for children with appendicitis and controls

	Controls (*N* = 3)	Appendicitis (*N* = 19)		
		Phlegmonous (*N* = 11)	Gangrenous (*N* = 4)	Perforated (*N* = 4)
Age (years)	2 (2–3)	12 (6–14)	9 (6–11)	8 (3–14)
Gender (M/F)	1/2	8/3	1/3	2/2
PAS	X	7 (4–10)	9 (7–10)	9 (7–10)
CRP value (mg/L)	X	13 (5–62)	50 (31–115)	231 (74–333)
Leukocytosis	X	8 (73)	3 (75)	4 (100)
Neutrophilia	X	8 (73)	3 (75)	4 (100)
Fever	X	7 (64)	4 (100)	4 (100)
Weight (kg)*	17 (15–18)	42 (20–87)	33 (22–44)	27 (15–44)
Fecalith	X	1 (9)	2 (50)	2 (50)

Values presented as median (min-max) or as the absolute number and percentage of patients; *n* (%)

*M* male, *F* female, *PAS* pediatric appendicitis score, *CRP* C-reactive protein

*All patients except one (phlegmonous appendicitis) had normal weight; one patient was obese (BMI > 30 kg/m^2^)

**Table 3:** Primer sequences for amplification of 16S rRNA genes, amplicon length 507 bp

16S Amplicon PCR Forward Primer	27F AGAGTTTGATCCTGGCTCAG
16S Amplicon PCR Reverse Primer	534R ATTACCGCGGCTGCTGG

*RNA ribosomal ribonucleic acid, *PCR* polymerase chain reaction

### Microbiome analyses

#### Phylum level

**Fig. 1:**
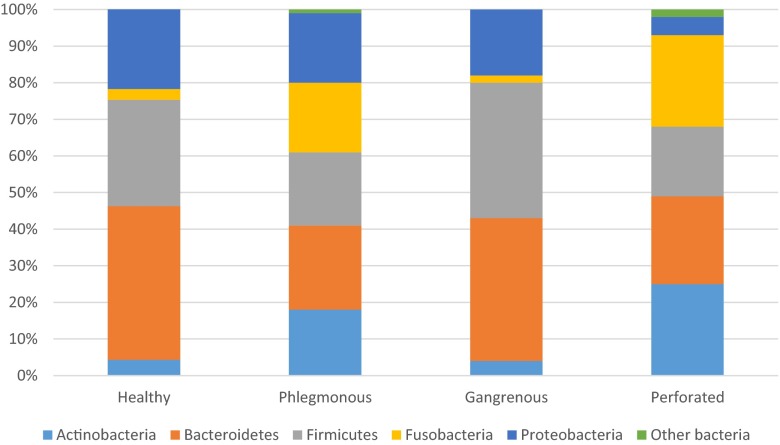
Microbiome analysis at phylum level of distal mucosa in patients with different grades of appendicitis compared with a control group. Phyla with a presence >2 % included in the figure

At the phylum level, ten different phyla were found. Five phyla were represented in all groups with a presence of >2 %, Bacteroidetes, Actinobacteria, Firmicutes, Fusobacteria, and Proteobacteria; whereas the phyla Spirochaetes, Cyanobacteria, Synergistetes, Tenericutes, and Verrucomicrobia, all had a presence of <2 % in all appendices. Bacteroidetes and Firmicutes were in majority among the controls (43 and 29 %, respectively). In phlegmonous appendicitis, there was an even distribution between the five phyla Actinobacteria, Bacteroidetes, Firmicutes, Fusobacteria, and Proteobacteria. Gangrenous appendicitis had an abundance of Bacteroidetes and Firmicutes (39 and 37 %, respectively), but low levels of Actinobacteria and Fusobacteria (4 and 2 %, respectively). Fusobacteria (25 %), Actinobacteria (25 %), Bacteroidetes (24 %), and Firmicutes were in abundance in perforated appendicitis (Fig. 1). No statistically significant differences in abundance at the phylum level described above were found (data not shown). When looking at the different phylum levels in patients within every separate group (e.g., different severity of appendicitis and controls), there was a wide variation of abundances within every specific group. Hence, patients with the same severity of appendicitis had very different levels of each specific phyla (data not shown).

#### Genus level

**Fig. 2:**
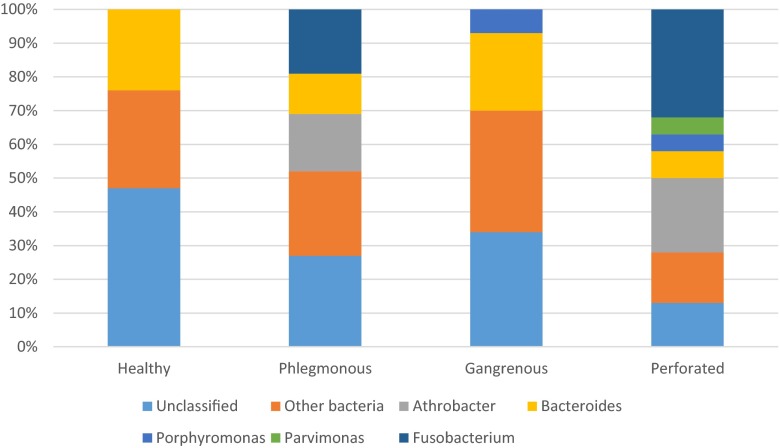
Microbiome analysis at genus level of distal mucosa in patients with different grades of appendicitis compared with a control group. Genus with a presence >5 % included in the figure

At the genus level, a total of 80 genera were found in the appendices. Only five genera had a presence of >5 %; *Athrobacter*, *Bacteroides*, *Porphyromonas*, *Parvimonas*, and *Fusobacterium* in any of the studied groups. In the controls, only *Bacteroides* (24 %) was present in >5 %. In phlegmonous appendicitis, *Fusobacterium* (19 %), *Athrobacter* (17 %), and *Bacteroides* (12 %) were in abundance. Gangrenous appendicitis was similar to the controls with *Bacteroides* having a major abundance (23 %), but with the addition of *Porphyromonas* (8 %) having an abundance of >5 %. In perforated appendicitis, five genus had a presence of >5 % with *Fusobacterium* (32 %) and *Athrobacter* (22 %) in majority (Fig. 2). No statistically significant differences in abundance at the genus level described above were found (data not shown). When looking at the different genus levels in patients within every separate group (e.g., different severity of appendicitis and controls), there was a wide variation of abundances within every specific group. Hence, patients with the same severity of appendicitis had very different levels of the different genera (data not shown).

Using the bioinformatics tool LEfSe, we further investigated whether appendicitis could be associated with any bacterial species, but there was no difference at species level between the groups, and as in the analyses at phylum and gender level, a wide variation was seen (data not shown).

#### Comparisons of microbiota according to histopathology

**Fig. 3:**
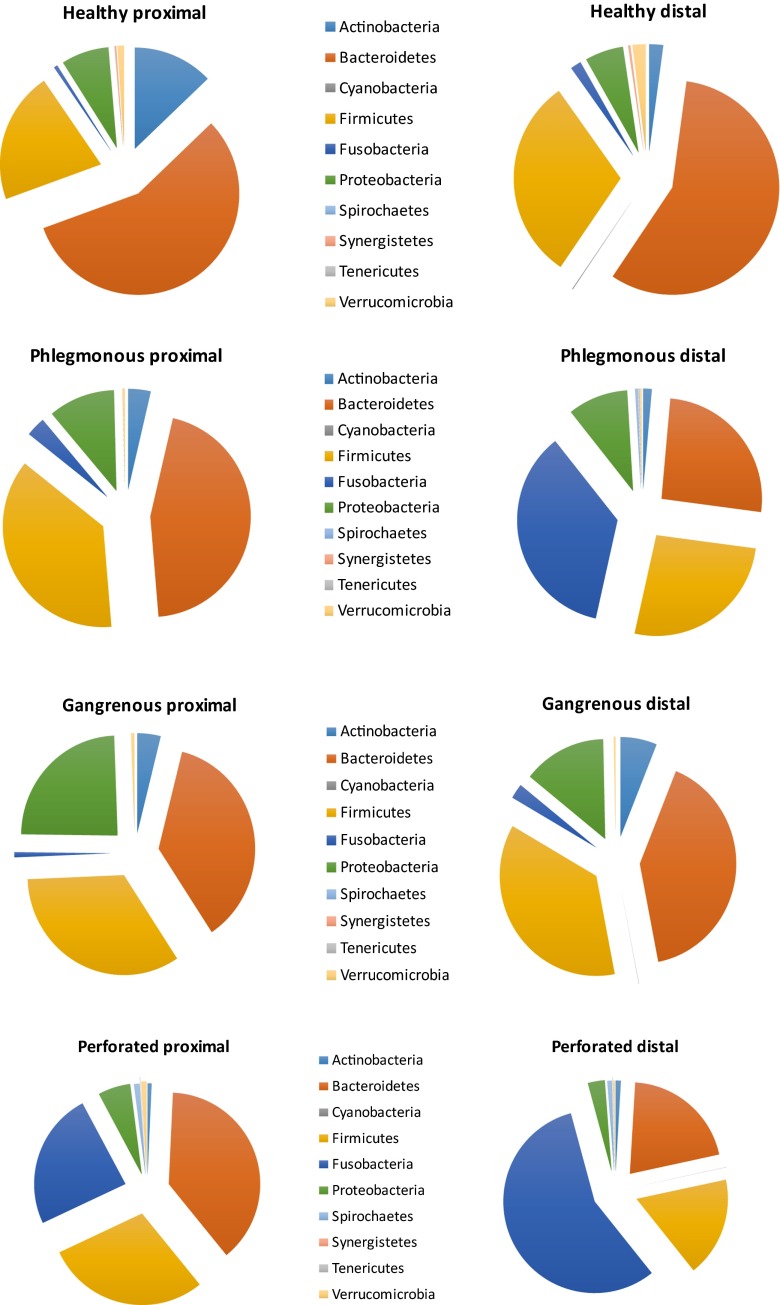
Microbiome analysis at phylum level of proximal and distal mucosa in different grades of appendicitis and controls

When comparing proximal mucosa and distal mucosa, no statistically significant differences were found at phylum or genus level (data not shown). At the phylum level in phlegmonous and perforated appendicitis, Fusobacteria had a presence in the proximal mucosa of 3 and 24 %, respectively, compared to 36 and 57 %, respectively, in the distal mucosa. The corresponding numbers for Bacteroidetes was 45 and 26 %, respectively, in the proximal mucosa, and 38 and 21 %, respectively, in the distal mucosa (Fig. 3).

There was no difference in phylum levels of the proximal mucosa between appendicitis patients with or without macroscopic inflammation at this site (data not shown). When comparing phylum levels of the distal mucosa between appendicitis patients with or without obstruction (appendicolith), there was a trend towards more abundance of Fusobacteria in patients with obstruction (25 and 13 %, respectively, *p* = 0.06). No differences were seen for other phyla (data not shown).

**Fig. 4:**
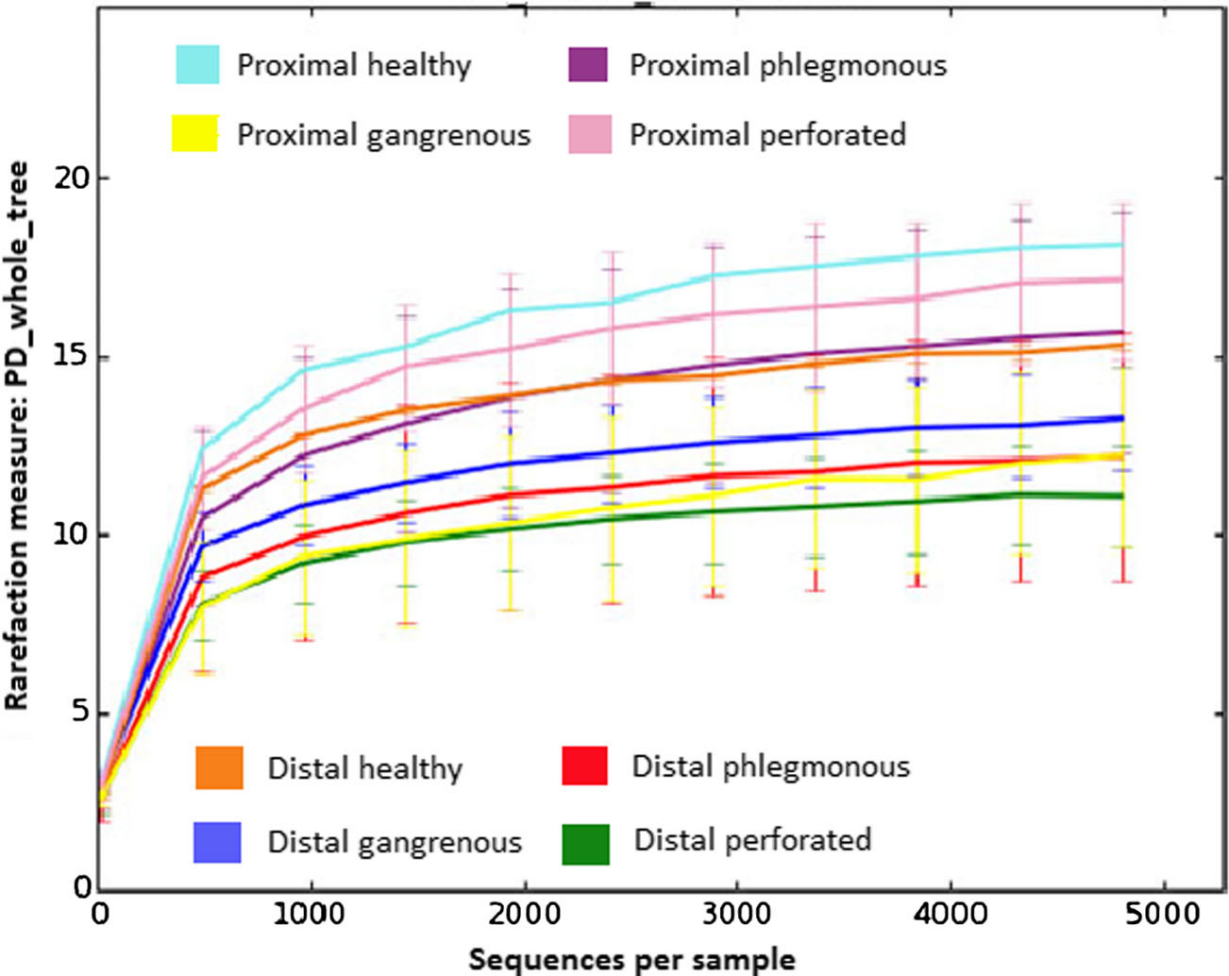
Alpha-diversity in patients with different grades of appendicitis and controls. Alpha-diversity was investigated with phylogenetic diversity (PD whole tree) and observed species (data not shown) indexes at an even sampling depth of 4831 sequences/sample. Values presented as mean ± SD. No significant differences between groups were found

No statistical significance was found when evaluating the taxa richness, but there was a trend with healthy appendices and proximal samples having higher α-diversity. Distal samples from perforated appendicitis had the least microbial diversity (Fig. 4). Unweighted and weighted Unifrac metrics did not show any significant clustering of the groups in a principal coordinate analysis (data not shown).

## Discussion

This was the first study evaluating the microbiome in all the three clinically defined stages of appendicitis and in relation to different sites and clinical conditions. At group level, distinct differences in phylum and genus abundances could be seen when comparing the controls with the three appendicitis groups. However, there was a wide variation of abundances within every specific group, which may explain the lack of significant differences seen throughout the study. There was also a variation in the microbiome between proximal and distal mucosa within each group. The only difference which tended to be statistically significant was the abundance of Fusobacteria distally to obstruction.

*Fusobacterium* increased in abundance in phlegmonous, and especially perforated appendicitis, compared to controls. However, it had a presence of <5 % in gangrenous appendicitis. The role of *Fusobacterium* in appendicitis has been described before [11, 15–18]. Swidsinski et al. [11] described *Fusobacterium* to be the cause of appendicitis in the majority of cases, which could not be confirmed in the present study. Among some of the present patients with phlegmonous and perforated appendicitis, *Fusobacterium* had a presence of <2 %. Several studies have described a correlation between the grade of inflammation and the presence of *Fusobacterium* [11, 15, 16]. However, compared to our study, no division of the appendicitis patients into the three clinically and histopathologically defined groups of appendicitis were made by others [11, 15, 16], and hence, no specific group with gangrenous appendicitis was analyzed. This may explain the difference in results compared to our study, where *Fusobacterium* decreased distinctly in gangrenous compared to phlegmonous appendicitis. Interestingly, we found that the patients with perforated appendicitis and high abundance of *Fusobacterium* also had a clear obstruction with the presence of an appendicolith. When comparing phylum levels between appendicitis patients with obstruction (appendicolith) and appendicitis patients without obstruction, there was a clear trend towards more abundance of Fusobacteria in patients with obstruction. As seen in the present study and other studies, bacteria from the phylum Fusobacteria seem to be a part of the normal appendix flora, but increased markedly in some of the cases of appendicitis [16, 18]. *Fusobacterium* is a part of the oropharyngeal flora and is also the most common oral anaerobe that give rise to infection outside the oral cavity [31]. Further, there has been reports on possible negative correlations between inflammatory bowel disease (IBD) and appendectomy [32, 33]. *Fusobacterium* and its degree of invasive potential have also been shown to be associated to inflammatory bowel disease (IBD) and to the IBD status of the host [34]. Also, alterations in the oral microbiome have been linked to pediatric IBD [35]. One might speculate that *Fusobacterium* could explain the link between appendectomy and IBD. In summary, with regard to the present and previous studies in the field [11, 15, 16, 18], bacteria from Fusobacteria may be a part of the pathogenesis in some but not the majority of the cases of appendicitis.

*Bacteroides* was, in the present study, abundant in not only healthy appendices, but also in gangrenous appendicitis. *Bacteroides* has been found to be inversely correlated to the degree of inflammation by some [18], but not others [16]. However, as stated above, the cited studies had no division of the appendicitis patients in to the three clinically and histopathologically defined groups of appendicitis, and hence, no specific group with gangrenous appendicitis was analyzed. This may explain the difference in results compared to our study, where *Bacteroides* increased distinctly in gangrenous compared to phlegmonous appendicitis.

Overall, when specifically reviewing every specific patient’s microbiome, it was clear that there was a wide variation of abundances at phylum, genus and species level, within every specific group, which also has been described by others [15, 18]. This, probably, not only explains the lack of significant differences between the groups in the present study, but also stresses the question of whether the microbiome plays a primary etiological role in the pathogenesis of appendicitis. Despite differences in phyla end genus between controls and appendicitis patients seen in this study and by others [11, 15, 16, 18], we can say that in many of the cases of appendicitis, bacteria do not seem to be the primary event. Rather, the differences in the microbiome are secondary events to another initial etiological factor, in parallel with the inflammatory cascade in the systemic immune defense. The findings may support the suspicion that appendicitis is of different etiology and the role of bacteria may be singled out only by excluding the situations where the appendicitis is for example due to an intraluminal obstruction only. Furthermore, it is a tempting thought that bacteriological findings may in the future influence the choice of treatment with antibiotics or an operative intervention.

When evaluating the microbiome, there are several possible confounders. Recent studies of the general population conclude the most important covariates to be medication, blood parameters, bowel habits, diet, health, anthropometrics, and lifestyle [36, 37]. All children were healthy and did not use any medication, alcohol, tobacco, coffee, or tea. Regarding age, gender, and weight, we found no correlation or differences in the microbiome; hence, all patients were calculated together. Their blood parameters correlated with the clinically and histopathologically defined grades of inflammation and were thus considered when different subgroups were compared. Regarding dietary habits, there is no present consensus of which type of food that affects the microbiome and how it affects. From recent publications, around 60 different dietary factors are proposed to have impact on the composition of the microbiome [37]. Further, there is evidence that the microbiome changes rapidly after changes in the diet [38]. Altogether, and with regard to the difficulty in making a thorough examination of dietary habits when dealing with an acute condition as appendicitis, it is impossible and probably too uncertain to try to adjust for dietary habits. At last, one would think that if appendicitis was driven by the microbiome, the bacteria composition should over-ride the differences in dietary habits.

A weakness of this study is the small population which may explain the lack of statistically significant differences, but there were more patients compared to the other studies of appendicitis with 16S rRNA sequencing [15, 16, 18]. Another weakness is that not all patients who underwent appendectomy during the study period were included; hence, the patients were included when the authors were on call. Therefore, a selection bias cannot be excluded. The strength of this study is that the study population was very homogenous with all children living in a small area, having the same ethnicity, and receiving identical preoperative antibiotic prophylaxis. Further, the microbiome was correlated to the three distinct clinical grades of appendicitis, which turned out to be important since gangrenous appendicitis had markedly different microbiome compared to phlegmonous and perforated appendicitis. Moreover, our study was the first to evaluate different parts of the mucosa and relating the microbiome to the macroscopically seen inflammation and presence of obstruction. Differences were seen, although significance was not reached, which is probably due to the low number of patients. Thus, since there may be differences between proximal and distal mucosa, it is important to describe the exact sample site of the appendix when evaluating the microbiome in future studies; otherwise, it is impossible to compare studies.

There is an ongoing discussion regarding the conservative treatment of appendicitis with antibiotics. In the light of such treatment, it is of great importance to fully understand the role of the microbiome in appendicitis. Hence, the differences in outcome reported may be due to patients with different microbial composition in the diseased appendix. Further studies of the microbiome in children with appendicitis should be performed, with larger study populations, and with description of possible obstruction of the appendix lumen and with exact sample site. Since bacteria normally present in the oropharyngeal flora was found to be in abundance in the patients with appendicitis, clinical data regarding oral infections, as well as sampling of the oral microbiome, could provide more information in future studies.

## Conclusion

The pattern of microbiome differed not only between groups, but also within groups. However, no statistically significant differences could be found in the microbiome between groups or clinical conditions. No correlation between a specific bacteria and grade of inflammation was found. In the vast majority of cases of appendicitis, changes in microbiome do not seem to be the primary event. Since there seem to be differences in microbiome patterns depending on sample site, the exact localization of biopsy sampling must be described in future studies.

### Contributors’ statements

Martin Salö: designed the study protocol, collected the clinical and laboratory data, drafted the initial manuscript, critically reviewed the manuscript, and approved the final manuscript as submitted.

Nittaya Marungruang: assisted in analysis and bioinformatical processing of the microbiota data, critically reviewed the manuscript, and approved the final manuscript as submitted.

Tiaa Sundberg: performed 16S rRNA gene sequencing of the microbiota, critically reviewed the manuscript, and approved the final manuscript as submitted.

Bodil Roth: performed 16S rRNA gene sequencing of the microbiota, critically reviewed the manuscript, and approved the final manuscript as submitted.

Pernilla Stenström: supervised the collection of data, critically reviewed the manuscript, and approved the final manuscript as submitted.

Einar Arnbjörnsson: designed the study, critically reviewed the manuscript, and approved the final manuscript as submitted.

Frida Fåk: assisted in bioinformatical processing of the microbiota data, critically reviewed the manuscript, and approved the final manuscript as submitted.

Bodil Ohlsson: designed the study, supervised the statistical calculations, critically reviewed the manuscript, and approved the final manuscript as submitted.

All authors approved the final manuscript as submitted and agreed to be accountable for all aspects of the work.
